# Gulf war illness: a tale of two genomes

**DOI:** 10.1186/s13104-024-06871-z

**Published:** 2024-08-21

**Authors:** Beatrice A. Golomb, Richard I. Kelley, Jun Hee Han, Bruce Miller, Leeann Bui

**Affiliations:** 1 Department of Medicine, University of California, San Diego, La Jolla, 92093 CA U.S.A.; 2 Department of Genetics and Genomics, Boston Children’s Hospital, 02115 Boston, MA U.S.A.; 3 Department of Obstetrics and Gynecology, Santa Clara Valley Medical Center, 95128 San Jose, CA U.S.A.

**Keywords:** Gulf war illness, Mitochondrial genetics, Nuclear genetics, Haplogroup DNA mutations, Oxidative stress

## Abstract

**Introduction:**

Gulf War illness (GWI) is an environmentally-triggered chronic multisymptom illness typified by protean symptoms, in which mitochondrial impairment is evident. It has been likened to accelerated aging. Nuclear genetics of detoxification have been linked to GWI.

**Objective:**

To see whether mitochondrial (mt) haplogroup U – a heritable profile of mitochondrial DNA that has been tied to aging-related conditions – significantly predicts greater GWI severity; and to assess whether GWI severity is influenced by mitochondrial as well as nuclear genetics. 54 consenting Gulf War veterans gave information on GWI severity, of whom 52 had nuclear DNA assessment; and 45 had both nuclear and mitochondrial DNA assessments. Regression with robust standard errors assessed prediction of GWI severity as a function of nuclear genetics (butyrylcholinesterase variants), mitochondrial genetics (haplogroup U, previously tied to aging-related conditions); or both.

**Results:**

BChE “adverse” variants significantly predicted GWI severity (β(SE) = 23.4(11.4), *p* = 0.046), as did mt haplogroup U (β(SE) = 36.4(13.6), *p* = 0.010). In a model including both, BChE was no longer significant, but mt haplogroup U retained significance (β(SE) = 36.7(13.0), *p* = 0.007). This is the first study to show that mitochondrial genetics are tied to GWI severity in Gulf-deployed veterans. Other data affirm a tie to nuclear genetics, making GWI indeed a “tale of two genomes.”

## Introduction

Gulf War illness (GWI) is a chronic, multisymptom fatiguing illness affecting ~ 1/3 of the ~ 700,000 U.S. troops deployed to the 1990–1991 Gulf theater. It is linked to chemical exposures, including acetylcholinesterase inhibitors [1] – organophosphates and pyridostigmine bromide. Culpable exposures produce mitochondrial toxicity, and GWI clinical features implicate mitochondrial compromise: protean symptoms of high multiplicity spanning multiple domains, emphasizing post-mitotic organs with high energy demand (brain and muscle) and variable symptom onset latency. Evidence affirms impaired bioenergetics in patients with GWI [2], while animal models of GWI further affirm mitochondrial compromise [3]. Nuclear genetic variants impairing natural detoxification mechanisms have been tied to GWI [4], including reduced activity variants of butyrylcholinesterase (BChE) in those with relevant exposures [5].

Mitochondrial genetics also warrant study. The maternally-inherited mitochondrial genome has only 37 genes, 13 protein encoding, but all relate to mitochondrial energy production and have an outsized impact on conditions related to cell energy. Mitochondrial haplogroups are heritable mitochondrial DNA variants, some of which are reported risk factors for genetic and non-genetic diseases and conditions. GWI has been likened to accelerated aging [6], and both GWI and aging are thought to involve the interplay of mitochondrial compromise and oxidative stress. Mitochondrial (mt) haplogroup U holds special interest, having been tied to increased risk of aging-related conditions in which these mechanisms are implicated, including Alzheimer’s disease, age-related hearing loss, and age-related maculopathy [7]. Therefore, we hypothesized that mt haplogroup U could be tied to greater severity of GWI.

## Materials and methods

The study was approved by the UC San Diego Human Research Protections Program, protocol number #151293. All participants gave written informed consent to participate. Of 54 consenting GWV who gave information on GWI severity, 52 had nuclear DNA assessment (23andMe), 47 had mitochondrial DNA assessment (GeneDx), and 45 had both. Each assessment of genetic prediction of GWI symptoms/severity employs the largest applicable sample (the maximum number of participants who underwent the requisite genetic assessments).

Nuclear DNA assessment: 23andMe version 5 genotyping employed Illumina’s Infinium Global Screening Array microarray platform, supplemented with ~ 50,000 variants of custom content [8]. Variant calling, by Illumina’s GenomeStudio software, employs probe intensities to assign genotypes to specific genetic variants. Reference SNP numbers (rs numbers) are assigned to each variant for unambiguous coding and provided by 23andMe to the investigator. All four BChE variants that contributed to the Steele 2015 analysis were available from 23andMe. No participants bore the F_1_ or F_2_ rare variants (rs28933389 and rs28933390) with respective U.S. minor allele frequencies of 0.083% and 0.47% [9]. One bore a copy of the atypical BChE variant (rs1799807) with estimated U.S. minor allele frequency of 1.6% [9]. The remaining slow-detoxifying variants came from the much more common K variant (rs1803274) with estimated U.S. minor allele frequency of ~ 20% [9], representing ~ 20% in our deployed and mostly Gulf War illness-affected sample.

Mitochondrial DNA assessment: Blood samples provided to GeneDx from participants underwent whole genome mitochondrial sequencing. Per GeneDx, mt haplogroup analysis was performed by GeneDx for each individual employing all homoplasmic mtDNA variants using an online tool. Phylotree.org was employed to “define the variants that determine haplogroup” [10]. “The average coverage (number of reads) for every nucleotide of the mitochondrial genome of a specific sample ranged from 5,000× to 25,000× for different samples, with a standard deviation of ≤ 10% of the average coverage. The coverage for nucleotide positions within 200 bp from the primer regions at the beginning and end of the mitochondrial genome could be lower due to removal of the primer sequences during alignment” [11].

GWI was characterized by CDC [12] and Kansas criteria [13]; and GWI severity was scored by the validated UCSD20 scale, which shows convergent validity with Kansas GWI criteria scores, physical function, general self-rated health, and correlates with impaired bioenergetics in veterans with GWI. BChE variant coding used Steele criteria [5] and was scored as 0, 1, or 2, and mt haplogroup U was scored as absent or present (0, 1). Regression with robust standard errors [14] assessed the prediction of GWI severity by mt haplogroup U and BChE variants separately, without and with adjustment for age. This was performed for all of the deployed participants, with subgroup analyses for males, whites, and white males – addressing the most common sex and ethnicity among those deployed. Regression then assessed prediction by mt haplogroup U adjusted for BChE genetic variants in these groups. Statistical output and database are available on request to the corresponding author.

## Results

54 consenting Gulf War veterans gave information on GWI severity. The 52 who had nuclear DNA assessment form the cohort for this study. Of these, 45 also had mitochondrial DNA assessment. Table 1 shows participant characteristics of 52 deployed Gulf War veterans: Most veterans were male, and White ethnicity predominated. Most met CDC and Kansas GWI criteria (*N* = 43), though severity varied.

**Table 1:** Participant characteristics

Variable*	Percentage
% Male	92.6
% White	74.1
% Married	83.3
% Haplogroup U	21.3
	**Mean (SD) [Range]**
Age (Years)	56.4 (7.48) [46,74]
# Kansas Domains (out of 6 possible)	4.19 (1.66) [0,6]
Summed Kansas Score	37.9 (20.0) [0,80]
UCSD GWI Symptom Number (of 20)	15.3 (5.51) [0,20]
UCSD GWI Symptom Score (of 200)	96.6 (50.1) [0,192]
BChE Score	0.404 (0.534) [0,2]

SD = standard deviation

*All 54 participants contributed to all assessments except haplogroup U (*N* = 47) and BChE (*N* = 52). 38.5% of participants had at least one contributory BChE variant

Table 2 shows prediction of GWI severity by mt haplogroup U and by BChE score assessed separately – in each of the four groups, without and with age adjustment. Both mt haplogroup U and BChE variants were significant predictors in all four groups, without and with age adjustment. Mt haplogroup U retained significance in regressions adjusted for BChE (all eight analyses), though significance was not upheld for BChE in the combined model. (There was no significant relationship between haplogroup U and the BChE variable.) Mt haplogroup U is primarily evident in European descended/White individuals, so for mt haplogroup U assessments, greatest validity attaches to analyses restricted to Whites. (Mt haplogroup U does occur, at much lower frequencies, in West Asia, North Africa, and parts of Central Asia, but no non-White participant in our sample bore this haplogroup.)

**Table 2:** Prediction of GWI severity by haplogroup U and BChE

			Haplogroup U			
		Without age adjustment		With age adjustment		
Group (Deployed)	N	β(SE) [CI]	P		β(SE) [CI]	P
All	47	36.4(13.6) [9.01,63.7]	0.010		34.0(12.3) [9.10,58.8]	0.009
Male	43	37.5(16.1) [4.97,70.1]	0.025		32.0(14.4) [3.03,61.0]	0.031
White	34	47.1(14.2) [18.2,76.0]	0.002		42.6(12.9) [16.3,68.8]	0.002
White, Male	30	49.3(16.9) [14.6,84.0]	0.007		40.1(14.9) [9.54,70.7]	0.012

			BChE			
		Without age adjustment		With age adjustment		
Group (Deployed)	N	β(SE) [CI]	P		β(SE) [CI]	P
All	52	23.4(11.4) [0.41,56.4]	0.046		23.1(11.4) [0.15,46.0]	0.049
Male	48	24.7(12.1) [0.29,49.2]	0.047		24.4(12.0) [0.17,48.6]	0.048
White	39	29.3(12.3) [4.43,54.2]	0.022		27.3(11.8) [3.39,51.3]	0.026
White, Male	35	31.7(13.2) [4.94,58.5]	0.022		29.3(12.5) [3.79,54.7]	0.026

			Haplogroup U adjusted for BChE			
		Without age adjustment		With age adjustment		
Group (Deployed)	N	β(SE) [CI]	P		β(SE) [CI]	P
All	45	36.7(13.0) [10.5,63.0]	0.007		35.1(12.2) [10.3,59.8]	0.007
Male	41	37.8(15.5) [6.33,69.2]	0.020		33.5(14.5) [4.14,62.9]	0.026
White	33	44.0(13.8) [15.8,72.2]	0.003		40.5(12.7) [14.6,66.5]	0.003
White, Male	29	45.7(16.6) [11.7,79.8]	0.010		37.6(14.6) [7.58,67.6]	0.016

β = regression coefficient; SE = standard error; CI = confidence interval

### Discussion & limitations

Our data, for the first time, implicate mitochondrial genetics in GWI. This adds to the corpus of support for mitochondrial involvement in GWI, already bolstered by both animal [3, 15–17] and human [2, 18, 19] data. When assessed separately, the designated mitochondrial and nuclear genetic factors (mt haplogroup U and BChE variants) each significantly predicted GWI severity, and each sustained significance in dominant sex- and ethnicity-subgroups, with and without age adjustment. Significance of prediction was sustained for mt haplogroup U, though not for BChE, in a model adjusting for both.

Limitations of the study include the modest sample size, which precluded assessment of gene-environment interactions. The robustness of the mt haplogroup U finding in sensitivity analyses coupled with other evidence supporting mitochondrial involvement in GWI, as well as evidence implicating mt haplogroup U in other conditions involving mitochondrial and oxidative mechanisms, bolster confidence in the observed mt haplogroup U relationship. Significance despite the modest sample, underscores the large apparent effect magnitude. (Conversely, loss of BChE variant significance in the mt haplogroup U adjusted model might be rectified with a larger sample: BChE variants were previously implicated in GWI [5].) A different study would be needed to assess whether mt haplogroup U is associated with accelerated development of these symptoms (presumably at a materially lower level) in persons not deployed to the Gulf: As most of us receive accrued exposures over time, and aging is associated with increased development of symptoms over time, we anticipate that analogous associations (albeit with much lower typical symptom levels) would be expected to arise in the general population – although a different study design would be required to assess this. Authority of findings will rest on replication, ideally in a larger sample. If possible, future longitudinal studies should evaluate whether and how mitochondrial haplogroup relates to ongoing GWI progression.

Since mt haplogroup U has been linked to accelerated aging conditions of other types, a question might be, what is the interplay between mt haplogroup U and GWI – alternatively said, to what extent would mt haplogroup U alone lead to such symptoms in the absence of Gulf War participation? The data here focus on individuals who were deployed, but we do have dates of first GWI-related symptoms for those participants who meet criteria for GWI. Almost all participants developed symptoms in the 1990’s, when most were in their 20’s – well before typical advent of aging-related symptoms. (Note that time since service in the Gulf is consistent (within a year) for participants, since Gulf War service dates are defined as between end of July 1990 and end of July 1991.) Mechanisms of mitochondrial impairment and accelerated oxidative stress arise from Gulf-related exposures, exposures that are significantly tied to development of GWI; it makes sense that a biological substrate that is more vulnerable to impairment, on its own or arising from exposures, would magnify GWI severity, given this evidence.

In conclusion, findings support the importance of mitochondrial genetics in GWI, and affirm that GWI is indeed a “tale of two genomes.” Implications are likely to extend beyond GWI – with anticipated relevance both to conditions promoted by environmental toxins, and to those entailing impaired bioenergetics.

## Data Availability

The datasets used and/or analyzed during the current study are available from the corresponding author on reasonable request.
